# Emerging Prospects for Combating Fungal Infections by Targeting Phosphatidylinositol Transfer Proteins

**DOI:** 10.3390/ijms22136754

**Published:** 2021-06-23

**Authors:** Danish Khan, Aaron H. Nile, Ashutosh Tripathi, Vytas A. Bankaitis

**Affiliations:** 1 Department of Biochemistry & Biophysics, Texas A&M University, College Station, TX 77843-2128, USA; danish@tamu.edu; 2 Calico Life Sciences LLC, 1170 Veterans Blvd, South San Francisco, CA 94080, USA; aaronnile@gmail.com; 3 Department of Molecular & Cellular Medicine, Texas A&M Health Sciences Center, College Station, TX 77843-1114, USA

**Keywords:** antifungal drugs, drug-resistant fungi, candidiasis, phosphoinositides, chemical genetics

## Abstract

The emergence of fungal “superbugs” resistant to the limited cohort of anti-fungal agents available to clinicians is eroding our ability to effectively treat infections by these virulent pathogens. As the threat of fungal infection is escalating worldwide, this dwindling response capacity is fueling concerns of impending global health emergencies. These developments underscore the urgent need for new classes of anti-fungal drugs and, therefore, the identification of new targets. Phosphoinositide signaling does not immediately appear to offer attractive targets due to its evolutionary conservation across the Eukaryota. However, recent evidence argues otherwise. Herein, we discuss the evidence identifying Sec14-like phosphatidylinositol transfer proteins (PITPs) as unexplored portals through which phosphoinositide signaling in virulent fungi can be chemically disrupted with exquisite selectivity. Recent identification of lead compounds that target fungal Sec14 proteins, derived from several distinct chemical scaffolds, reveals exciting inroads into the rational design of next generation Sec14 inhibitors. Development of appropriately refined next generation Sec14-directed inhibitors promises to expand the chemical weaponry available for deployment in the shifting field of engagement between fungal pathogens and their human hosts.

## 1. Introduction

The human population carries a remarkable load of commensal microorganisms that execute functions beneficial to the human host. These benefits include symbiotic provision of micronutrients to the host, occupancies of ecological niches that are otherwise conducive to invasion by pathogenic microorganisms, and a consistent low-level priming of the immune system that readies the host to respond to infection [1,2]. While superficial fungal infections in humans are commonplace, overgrowth of commensal fungi can develop into life-threatening systemic infections. Such deep fungal invasions represent an escalating health problem worldwide and claim in excess of 1.5 million lives annually [3,4]. Moreover, these diseases come with mortality rates surpassing those associated with many viral and bacterial infections [5]. These metrics are realized even in the face of treatment. For example, the mortality rates associated with *Aspergillus fumigatus* and disseminated mucormycosis infections approach 100% in undiagnosed cases and are as high as 50% in cases that are diagnosed and treated [5,6,7,8].

Greater than 90% of the reported deaths due to fungal disease are attributed to infection by *Aspergillus*, *Candida*, *Cryptococcus,* and *Pneumocystis* species [5,9,10,11]. Although prevention is the best medicine in terms of fungal infection control, management becomes a serious problem once infection takes hold—as the mortality rate numbers report. Thus, one of the challenges presently facing infectious disease clinicians is that successful management of invasive fungal infections requires effective intervention during the early stages [12,13,14]. Herein, we briefly discuss the current status of antifungal drug therapy and the urgent need for development of new tools for chemical intervention. This urgency is fueled by the rapidly climbing incidence of human fungal diseases, coupled with the rise of multi-drug-resistant fungal pathogens. In this review, we focus on future prospects for expanding the dwindling antifungal drug arsenal with primary emphasis on targeting phosphoinositide signaling, specifically, PITP-dependent phosphatidylinositol 4-phosphate [PtdIns(4)P] signaling in these pathogens. While the discussion focuses on *Candida* as infectious agent, these concepts translate to other fungal pathogens as well.

## 2. Virulence of *Candida* Species

**Figure 1:**
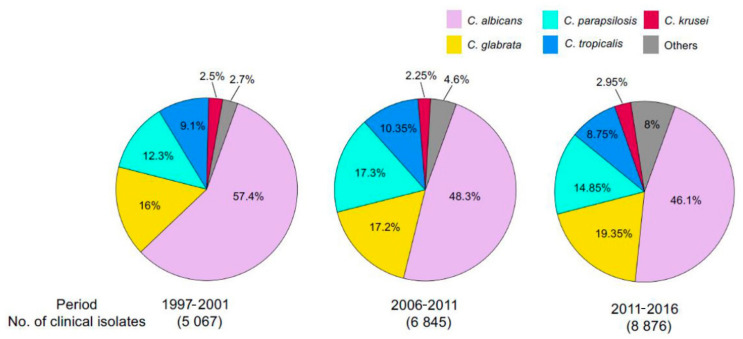
Distribution of Candida spp. in cases of invasive candidiasis. Species distribution in cases of invasive candidiasis over a period of 20 years is shown as percentage of total. Clinical isolates of candidemia were collected from hospitals in 41 countries as part of the global SENTRY Antifungal Surveillance Program and *Candida* spp. identified. Total number of clinical isolates for a given time period is given in parenthesis. Adapted from ref. [21].

Of the approximately 20 virulent *Candida* species, isolates obtained from candidemia patients are dominated by five species—*C. albicans*, *C. glabrata*, *C. krusei*, *C. parapsilosis,* and *C. tropicalis* [15,16,17]. Of these, *C. albicans* infections account for approximately half of all infections in the USA, but serious threats are now arising from other *Candida* species as well (Figure 1). For example, the incidence of *C. glabrata* infection has steadily climbed over the past decade and currently accounts for some 33% of total infections [18,19]. Infection by different fungal pathogens is accompanied by varied strategies by which these organisms circumvent the host immune system. For example, *C. albicans* transitions to a filamentous growth mode that produces a hyphal mycelium that is extremely difficult to clear once established. By contrast, *C. glabrata* does not progress through a mycelial phase and behaves more like its non-pathogenic cousin *Saccharomyces cerevisiae* [20].

*Candida* populate the human gastrointestinal tract as components of the normal gut microbiota [22,23]. The switch from commensal to opportunistic behavior occurs when host immunity is weakened or when the mucosal barrier is mechanically breached. Patients undergoing hemodialysis, organ transplants, or other surgical procedures are especially vulnerable—as are patients with compromised immune systems, such as the elderly, newborns, patients with viral infections (e.g., HIV, COVID-19), and cancer patients undergoing chemotherapy. The primary sources of nosocomial candidiasis are intravascular catheter tubes contaminated with fungal biofilms [24,25]. Invasive candidiasis includes bloodstream infections, as well as candidiasis of organs—particularly kidney, liver, spleen, eye, or lung [13,26,27,28,29]. As the incubation period between exposure of the patient to *Candida* and onset of fulminant sepsis is typically 7 to 10 days, the window of opportunity for effective intervention is uncomfortably tight [28,30].

## 3. Therapeutic Options for Treating Candidiasis

**Figure 2:**
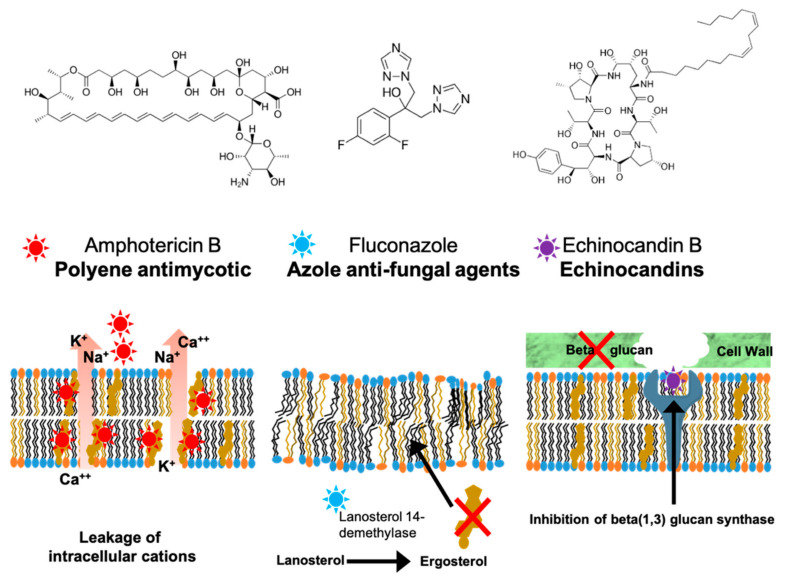
The major classes of antifungal drugs used in the clinic and their mechanisms of action. Panel illustrates available classes of anti-fungal therapeutics, polyenes (**left**), azoles (**middle**), and echinocandins (**right**). Their corresponding mechanisms of action are illustrated at bottom.

Despite their relatively small genomes, *Candida* species exhibit significant phylogenetic relationships with humans, as evidenced by these fungi encoding homologs for about 30–50% of human genes [31]. This similarity demands careful selection of fungal targets for pharmaceutical intervention as many of the druggable targets in *Candida* have human counterparts—thereby increasing the likelihood of undesirable toxicities associated with off-target effects. It is partially for this reason that therapeutic strategies for combating invasive candidiasis primarily revolve around three US Food and Drug Administration (FDA) approved classes of molecules—the polyenes, azoles, and echinocandins [32,33,34]. Collectively, these antibiotics interfere with two distinct activities in fungi. Polyenes and azoles are sterol-active compounds that exploit the chemical differences between host and fungal sterols (cholesterol and ergosterol, respectively; Figure 2. Polyenes disrupt membrane sterol organization, while azoles inhibit sterol synthesis [35]. Echinocandins interfere with fungal cell wall biosynthesis.

Polyenes, such as the natural product amphotericin B, represent some of the oldest anti-fungals (Figure 2. Amphotericin B is produced by soil actinomycetes, and polyenes exert their pharmacological effects by binding the major fungal sterol ergosterol in the plasma membrane with sub-micromolar dissociation constants [36]. The binding reaction promotes assembly of pores of various sizes, including large conductance pores which perforate fungal plasma membranes and kill the pathogen [36,37]. The reduced affinities of polyenes for cholesterol, the bulk mammalian sterol, as compared to the higher affinities of polyenes for the fungal sterol ergosterol, form the basis for the relative fungal selectivity of these compounds [36]. Unfortunately, polyenes come with serious disadvantages, such as poor bioavailability and the propensity of polyenes to induce significant off-target toxicities—primarily nephrotoxicities [38,39]. These side-effects reflect the fact that the fungal ergosterol and host cholesterol, while chemically distinct lipids, are nonetheless structurally similar molecules. Thus, the differential binding affinities of polyenes for ergosterol relative to cholesterol are insufficient to ensure optimal target specificity. Whereas emerging technologies strive to improve anti-fungal potencies by complexing new generation polyenes with lipid mixtures, undesirable toxicities remain problematic [40,41,42].

The last three decades witnessed the rise of azoles (e.g., fluconazole, triazole) as drugs of choice to treat fungal infections (Figure 2). Azoles target ergosterol biosynthesis by inhibiting the lanosterol 14-alpha-demethylase (product of the *ERG11* gene) at the endoplasmic reticulum [35,43]. These drugs reduce the ergosterol content of the fungal plasma membrane and induce a number of perturbing effects that include compromise of plasma membrane rigidity. While azoles show excellent pharmacokinetic properties, their extensive use applies a selection pressure for clinical isolates exhibiting an ever-increasing resistance to these compounds [44,45].

Echinocandins are inhibitors of the β-1,3-D glucan synthase complex encoded by the fungal *FKS* genes [46,47,48] (Figure 2). As such, these compounds inhibit cell wall biosynthesis and can be broadly viewed as “fungal penicillins” [49]. Unfortunately, echinocandin therapy is plagued by the poor bioavailability of these compounds. Moreover, anidulafungin, the last antifungal developed from the echinocandin class, was approved by the FDA more than a decade ago [49,50]. Progress is being made in that regard, however, as the new echinocandin-class drug rezafungin shows promise in treatment of candidiasis in mouse models [51]. Another echinocandin class drug, ibrexafungerp, is being developed by Synexis, Inc and is in phase III clinical trials [52,53].

## 4. Multidrug-Resistant Fungal Pathogens

The heavy clinical reliance on polyenes, azoles, and echinocandins as anti-fungals is inexorably driving the emergence of drug resistant *Candida*. While some *Candida* (e.g., *C. kruzei*) are intrinsically azole-resistant, excessive prophylactic use of fluconazole has selected for azole resistance in previously sensitive species. For example, *C. albicans* and *C. glabrata* isolates resistant to the third-generation azole voriconazole have already appeared [11,54]. Although amphotericin B resistance has historically been rare, polyene-insensitive isolates of *C. albicans*, *C. glabrata*, and *C. tropicalis* are also emerging [55,56,57]. The health crisis caused by emergence of multi-drug resistant isolates of the Gram-positive bacterium *Staphylococcus aureus* (MRSA) offers a chilling preview of what the advent of multi-drug resistant *Candida* species will bring to the infectious disease arena [58,59,60]. In that regard, the rapid emergence of *Candida auris* as an infectious agent of deep concern reflects the graduation of this possibility to reality [19,61,62,63].

*C. auris* was first identified in Japan in 2009, and this pathogenic fungus is disseminated primarily via nosocomial infections [61]. Unlike other *Candida* species that typically reside in the gastrointestinal tract, *C. auris* biofilms efficiently colonize the skin of immunocompromised patients [64]. In the year 2020, 714 clinical cases of *C. auris* infection were documented in the USA [65]. Because traditional methods of fungal phenotyping routinely mis-identify *C. auris* as either *C. haemuloni* or *C. famata*, this statistic is almost certainly an underestimate. A particularly alarming development is that most *C. auris* isolates in the USA not only present complete resistance to azoles but ~30% of those isolates are amphotericin B resistant as well [63]. That leaves treatment of *C. auris* infections with echinocandins as the sole option. Unfortunately, some 2% of all clinical isolates of *C. auris* in the United States are already echinocandin resistant [66]. Thus, *C. auris* is on the cusp of evolving into a eukaryotic superbug impervious to all available anti-fungals. Given that *C. auris* has spread to more than 30 countries to date, and that its global dissemination is likely to continue at a rapid pace, the CDC designates this pathogen as a “serious global health threat” [65].

## 5. New Targets and Next Generation Anti-Fungal Compounds

Combating the threat of emerging fungal pathogens requires the development of next generation anti-fungals. One approach towards that goal is to maintain focus on familiar targets but to intervene with those target activities in new ways. Effective management of emerging fungal pathogens also demands greater urgency in development of new drugs that target unappreciated biological activities, as it is in this arena where novel opportunities are to be found. We anticipate this general approach will include pharmacological impairment of activities conserved between pathogen and host that, upon first inspection, would seem to invite undesirable off-target toxicities. However, emerging evidence indicates that such strategies might prove effective. The main topic of this review focuses on precisely this topic. We explore the essentially undiscussed prospect of intervening with fungal phosphoinositide signaling as a strategy for producing new lead compounds for development of next-generation antimycotics. To that end, we first introduce the cell biology and enzymology of phosphoinositide synthesis. Second, we describe the critical non-enzymatic role a poorly understood class of proteins, namely, the Sec14-like phosphatidylinositol transfer proteins (PITPs), play in stimulating the activities of lipid kinases that drive phosphoinositide production and signaling. Finally, we describe the experimental data that identify PITPs as attractive targets for highly specific chemical inhibition of fungal phosphoinositide signaling, and we outline the opportunities these afford for development of next-generation anti-fungal drugs.

## 6. Phosphoinositide Signaling

**Figure 3:**
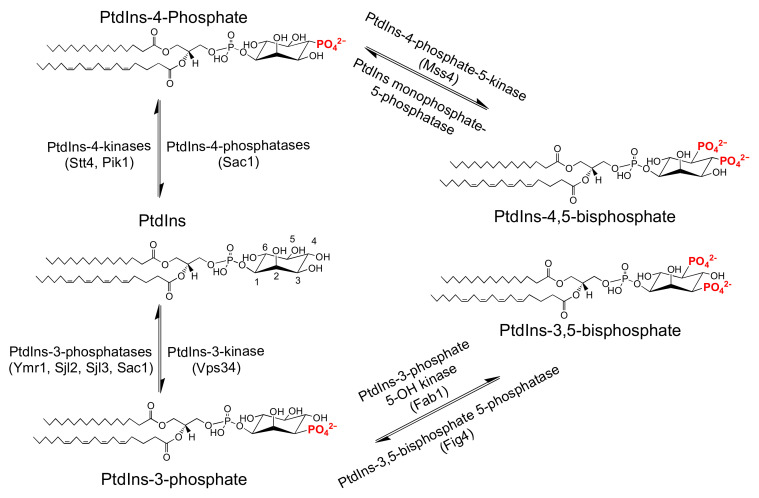
S. cerevisiae phosphoinositide metabolism. Along with phosphatidylinositol, yeast produces five distinct phosphoinositide species. Synthesis of individual phosphoinositide species occur by one or two sequential phosphorylation events at the phosphatidylinositol headgroup. All phosphoinositide species are generated by the action of specific kinases, with the exception of PtdIns(4)P, whose production is catalyzed by one of two distinct and functionally non-redundant PtdIns 4-OH kinases. PITPs boost the formation of PtdIns(4)P by priming the activities of both of these PtdIns 4-OH kinases. Phosphoinositide phosphatases act in opposition to the lipid kinases to degrade phosphoinositides. Some of these enzymes exhibit somewhat relaxed substrate specificities. For example, Sac1, the major PtdIns(4)P phosphatase in yeast, has the capacity to catalyze dephosphorylation of several mono- and bis-phosphoinositide species.

Phosphatidylinositol (PtdIns) is an essential phospholipid in eukaryotes as it represents the metabolic precursor for a set of chemically distinct phosphorylated derivatives -the phosphoinositides. The chemical uniqueness of each phosphoinositide is defined by the specific position(s) at which the inositol (Ins) headgroup of PtdIns is phosphorylated. These modifications, either singly or in combination, involve the 3-OH, 4-OH, and/or 5-OH positions of the Ins ring (Figure 3). The synthesis of each phosphoinositide is governed by the activities of positionally-specific PtdIns kinases [67,68,69,70]. Their degradation is catalyzed by phosphoinositide phosphatases and by phospholipases that exhibit some measure of positional-specificity [67,71,72,73].

The biological essentiality of phosphoinositides reflects the versatility of these lipids in controlling various aspects of signal transduction in eukaryotic cells. The intrinsic chemical diversities of phosphoinositides represent the foundation upon which a highly diverse intracellular chemical signaling code is built [74,75,76,77]. Phosphoinositides hold intrinsic signaling power as these lipids promote spatially and temporally controlled recruitment of effector protein activities from the cytoplasm onto chemically defined platforms on membrane surfaces. These lipids are also critical cofactors for the activities of other key signaling proteins, such as phospholipase D [78,79,80] and ion channels [81,82].

Phosphoinositide cleavage by phospholipases C produces both soluble and lipid second messengers. In the case of phospholipase-C-mediated hydrolysis of PtdIns-(4,5)-bisphosphate [PtdIns(4,5)P_2_], this reaction produces both a signaling pool of the neutral lipid diacylglycerol that activates protein kinases C and a soluble pool of Ins-(3,4,5)-trisphosphate that launches waves of calcium signaling [83,84]. Soluble Ins-polyphosphates and Ins-pyrophosphates derived from Ins-(3,4,5)-trisphosphate are themselves critical players in other cellular functions. These include signal transduction activities executed via allosteric control of protein activities [85,86,87,88]. Not all roles involve signaling—at least not as classically defined. In some cases, soluble Ins phosphates are structural co-factors obligatorily required for protein folding and/or stability [69,89].

## 7. PtdIns(4)P Synthesis and Signaling

The phosphoinositide of focus in this work is PtdIns(4)P or, in other words, PtdIns modified with a single phosphate at the 4-OH position of the Ins ring. This lipid executes its own dedicated set of signaling activities in the cell in addition to serving as metabolic precursor for PtdIns(4,5)P_2_. PtdIns(4)P integrates a wide swath of the lipid metabolome with the activity of the trans-Golgi network (TGN) and endosomal membrane trafficking machinery. This phosphoinositide is the product of PtdIns 4-OH kinase (PI4K) activity, and PI4Ks fall into two distinct structural classes categorized as type II enzymes (PI4KIIα, PI4KIIβ) and type III enzymes (PI4KIIIα, PI4KIIIβ) [70]. These enzymes localize to different intracellular compartments and, as such, produce spatially distinct PtdIns(4)P pools. These PI4Ks are functionally non-redundant enzymes, as both PI4KIIIα and PI4KIIIβ are each individually essential for yeast cell viability.

The biological non-redundancy of PI4Ks reflects the functional distinction of the respective PtdIns(4)P pools produced. PI4KIIIα and PI4KIIIβ activities (Stt4 and Pik1, respectively) account for the majority of the PtdIns(4)P produced by yeast [68], and these enzymes generate plasma membrane and TGN/endosomal pools of PtdIns(4)P, respectively [68,90,91]. The TGN/endosomal pools of PtdIns(4)P are essential for viability of eukaryotic cells because this phosphoinositide regulates the activities of many pro-membrane trafficking factors. These include small GTPases and their effectors [92,93,94], lipid flippases and cargo adaptors [95,96,97,98,99,100,101,102,103,104], coat protein adaptors involved in recruitment of clathrin to membranes [105,106], and protein kinases involved in Golgi-derived transport vesicle biogenesis [107,108]. These trafficking involvements also manifest themselves in decidedly non-canonical cellular contexts as Golgi-derived vesicles containing PtdIns(4)P are now reported to control mitochondrial morphology by promoting fission of this organelle at mitochondrial/endoplasmic reticulum membrane contact sites [109].

## 8. PtdIns(4)P Signaling and the PI4K-PITP Partnership

At first glance, targeting PtdIns(4)P signaling for pharmacological intervention would seem an unlikely strategy given the conserved nature of PtdIns(4)P signaling and the homologies shared by fungal and mammalian PI4Ks that are the obvious targets for such intervention. However, we now understand there are effective and highly specific ways to interfere with PI4K signaling without targeting the enzymes directly. This curious opportunity stems from the under-appreciated vulnerabilities of these enzymes. A unique feature of cellular control of PtdIns(4)P homeostasis by PI4KIIIα and PI4KIIIβ is that both PI4K enzymes, by themselves, are incapable of generating sufficient PtdIns(4)P to overcome the activities of PtdIns(4)P signaling antagonists and elicit biological responses. To do so, PI4Ks require the activities of PtdIns transfer proteins (PITPs) that potentiate PI4K activities so that sufficient PtdIns(4)P is generated to promote signaling in the face of the opposing action of erasers of PtdIns(4)P signaling [74,110,111,112].

**Figure 4:**
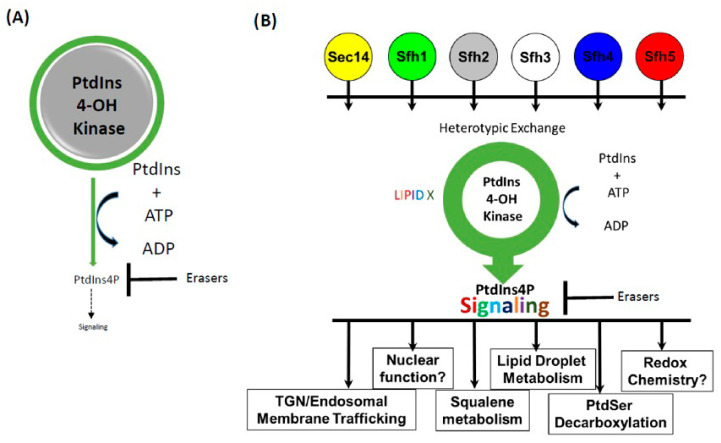
PITPs as novel portals for surgical intervention with specific channels of PtdIns(4)P-signaling. (**A**) PtdIns 4-OH kinases are intrinsically inefficient enzymes in physiological settings. These enzymes are incapable of generating sufficient PtdIns(4)P to overcome the activities of antagonists (erasers) of PtdIns(4)P signaling (e.g., phosphoinositide phosphatases). (**B**) Sec14-like PITPs potentiate PtdIns 4-OH kinase activities so that these enzymes produce sufficient PtdIns(4)P for signaling in a temporally and spatially regulated manner. Sec14-like PITPs specify metabolic inputs into production of unique PtdIns(4)P pools that promote unique biological outcomes. These are proposed to do so by engaging in transient complexes where an individual PITP interacts with a PtdIns 4-OH kinase in the context of a specific set of PtdIns(4)P effectors, assembled either as individual proteins or in PITP multidomain arrangements. The identity of the PITP in the complex specifies the metabolic input in the form of the second ligand they bind for priming PtdIns presentation to the PtdIns 4-OH kinase, and the cohort of proximal PtdIns4P effectors determines biological outcome.

In terms of mechanism, PITPs help the biologically insufficient PI4K solve an interfacial catalysis problem via the coupling of a heterotypic exchange cycle involving PtdIns and a second lipid ligand that allows the PITP to “present” PtdIns to the PI4K (Figure 4). This heterotypic exchange cycle occurs on the membrane surface and PITP-driven “presentation” of the PtdIns molecule renders the engaged PtdIns a superior substrate for the PI4K. The PtdIns presentation model posits that evolution takes advantage of the Sec14-fold to produce a battery of modules that collectively bind PtdIns, along with a large variety of second lipid ligands. This biochemical diversity is then translated into a diverse array of biological outcomes for signaling from an otherwise homogeneous PtdIns(4)P chemical code [74,110]. It also conceptualizes “signaling pixels” as integrated PITP:PI4K-effector circuits [111,112,113]. Thus, PITPs offer a portal through which PI4K signaling can be drugged with high specificity without targeting PI4K enzymes themselves.

The remainder of this article focuses on describing the mechanism for how Sec14-like PITPs potentiate PI4K signaling and outlines the body of evidence that identifies PITPs as attractive targets for the development of next-generation anti-fungals. The evidence indicates that fungal Sec14-like PITPs and, by extension, fungal phosphoinositide metabolism and membrane trafficking are unexpectedly attractive targets for small-molecule-mediated inhibition [50,114,115,116,117]. Recent progress on these fronts identifies new avenues for pursuing the design of improved inhibitors for use as lead compounds for development of next-generation anti-fungal drugs.

## 9. Mechanisms of PITP Function

PITPs constitute an ancient family of proteins that are present throughout the *Eukaryota*. These enigmatic proteins fall into two evolutionarily distinct groups based on primary structure and structural considerations—PITPs of the Sec14-fold and those of the steroidogenic acute response-related transfer (StAR) domain family [111,112]. The Sec14-like PITPs share homology with the *S. cerevisiae* Sec14—the founding member of the Sec14-domain protein superfamily [118,119]. Despite their structural unrelatedness, PITPs of both the Sec14-like and StAR families stimulate PI4K activities in vivo [102,120,121]. Because fungal pathogens express Sec14-like PITPs, the focus will be on PITPs of this superfamily.

**Figure 5:**
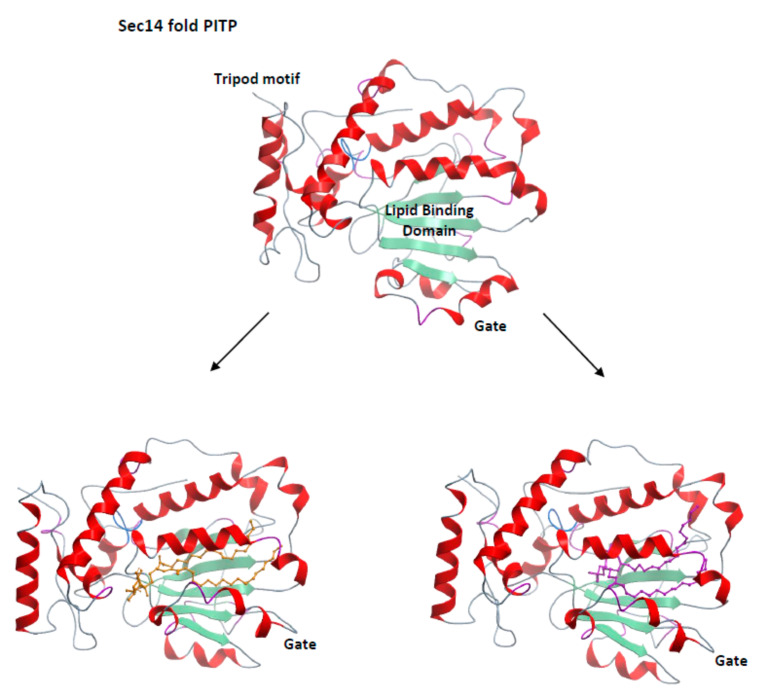
Structural features of Sec14-like PITPs. The Sec14 structural fold is depicted in ribbon diagram. This compact fold consists of two domains, the N-terminal tripod motif and the lipid binding domain whose lipid-binding cavity floor is defined by four parallel β-strands. At top is shown the structure of the lipid-free apo form that describes the open conformer (PDB: 1AUA) where the gate that controls access to the Sec14 lipid binding pocket is swung open. At bottom left and bottom right are depicted PtdIns- (orange ball&stick render) and PtdCho (magenta ball&stick render)-bound holo-forms of the Sec14-fold in the closed conformation, respectively. In both holo forms, the gate has undergone an ~18Å conformational shift to obstruct access to the lipid binding pocket. These holo structures are of a close *S. cerevisiae* paralog of Sec14 (Sfh1) and the high resolution PtdIns- and PtdCho-bound structures are described in PDB: 3B7N and PDB: 3B7Q, respectively.

As discussed above, Sec14-like PITPs are not enzymes as these molecules do not have the capacity to chemically modify their binding substrates by themselves. Rather, these proteins behave as “nanoreactors” that leverage a heterotypic lipid exchange cycle to “present” PtdIns molecules to PI4K on a membrane surface—thereby boosting PI4K activity [74,110,122,123]. From the structural point of view, the Sec14-fold is a compact design characterized by a large lipid-binding pocket and an α-helix that gates entry to the pocket and undergoes large conformational motions during the transitions between “closed” and “open” conformers [110,124,125] (Figure 5). It is in the context of these conformational rearrangements that heterotypic exchange of PtdIns and the second lipid ligand, in the case of Sec14 phosphatidylcholine (PtdCho), occur. The fact that the PtdIns and PtdCho headgroup binding motifs are spatially well-separated within the Sec14 lipid-binding cavity gives rise to the interesting proposition that “PtdIns-presentation” involves abortive attempts by PtdIns to invade a Sec14 pocket that is still occupied by PtdCho [74,110].

Interestingly, the PtdIns-binding barcode is conserved throughout the Sec14 superfamily, whereas the PtdCho-binding barcode is divergent [74,110,113,126]. Even in *S. cerevisiae*, two of its Sec14-like PITPs (Sec14 and Sfh1) are PtdIns/PtdCho-exchange proteins while the remaining four are not [110,127,128,129,130]. (Figure 5). Sfh2 and Sfh3 are PtdIns/squalene and PtdIns/ergosterol exchange proteins, respectively [131], whereas the biological function of Sfh4 in stimulating phosphatidylserine decarboxylation in endosomes does not require its PtdIns exchange activity. Rather, Sfh4 serves as a non-catalytic subunit of the decarboxylase. This Sfh4 activity can be uncoupled from its activity as a PITP and its in vivo ability to stimulate PI4K activity [129]. Finally, Sfh5 is a novel heme-binding protein that is not designed to function as a PITP at all [130]. It is the heterogeneities in the chemical properties of the lipid binding pockets of Sec14-like PITPs that determine the specificity of Sec14 targeting by small molecule inhibitors.

## 10. Sec14 Orthologs in Virulent Fungi

*S. cerevisiae* Sec14-like PITPs all execute distinct functions in vivo, and Sec14 executes an essential function required for yeast cell viability [118]. While Sec14 potentiates the activities of both yeast PI4KIIIα and PI4KIIIβ enzymes [114], its essential function is to integrate various aspects of lipid metabolism with PtdIns(4)P signaling in the TGN/endosomal system of yeast [67,71,132,133]. That activity is not only required for protein transport from the TGN/endosomal system to the vacuole and to the cell surface [118,134] but it also interfaces with cell cycle control [135]. Early studies indicated that Sec14 orthologs of dimorphic fungi, such as *Yarrowia lipolytica* and *C. albicans*, are required for proper execution of transitions from yeast-type budding cell divisions to the filamentous growth mode [136,137,138]. This Sec14 function is of interest because transition to the hyphal form is critical to formation of a mycelium and, therefore, for the pathogenesis of many fungal species [137,139]. While Sec14 activity is essential for *C. albicans* viability, even reduced Sec14 activity is incompatible with this organism forming hyphal filaments—with the result that virulence is strongly compromised [137]. The major morphological transition from yeast budding mode of cell division to the filamentous mode pose attractive biological targets for chemical inhibition given this transition is a complex process marked by widespread reorganization of the fungal metabolome [140].

A critical role for Sec14 has also been documented for the opportunistic pathogen *Cryptococcus neoformans*—a causative agent of lung infections that frequently progress to cryptococcal encephalomeningitis in immune-compromised individuals. This infection represents a leading cause of death in AIDS patients [141]. The virulence of this organism is dependent upon its ability to secrete extracellular virulence factors, such as cryptococcal phospholipase B1 (CnPlb1) and the Mpr1 metalloprotease, into its host. Secreted Mpr1 is required for *C. neoformans* invasion across the blood–brain barrier [142], whereas CnPlb1 is a multifunctional secretory protein that hosts multiple enzymatic activities—all of which are geared towards mobilization of fatty acids from host membranes and to the diminution of host immune responses [143]. Extracellular CnPlb1 activity is required for infection of host phagocytes by *C. neoformans*, a process that helps the pathogen evade the immune response and invade the central nervous system. Moreover, secretion of this virulence factor from the pathogen is Sec14-dependent [139]. Whether secretion of other cryptococcal virulence factors is also Sec14-dependent is unknown but remains a likely possibility. Taken together, there is ample biological rationale to consider fungal Sec14-like PITPs as attractive targets for next-generation anti-fungal drugs.

## 11. Targeting Fungal Phosphatidylinositol-4-Phosphate Signaling by Exploiting the PI4K-PITP Partnership: First Generation Sec14 Inhibitors

Although an obvious way to interfere with PtdIns(4)P signaling is to target PI4K for inhibition, this strategy comes with potential disadvantages [144,145]. One is that the catalytic sites of these enzymes, and kinases in general, are highly conserved, and inhibitors directed against fungal PI4Ks are likely to come with undesirable off-target effects. It is from this perspective that chemical interference with PITP-mediated instructive channeling of PtdIns(4)P synthesis provides novel opportunities for interfering with PtdIns(4)P signaling in a far more surgical way.

**Figure 6:**
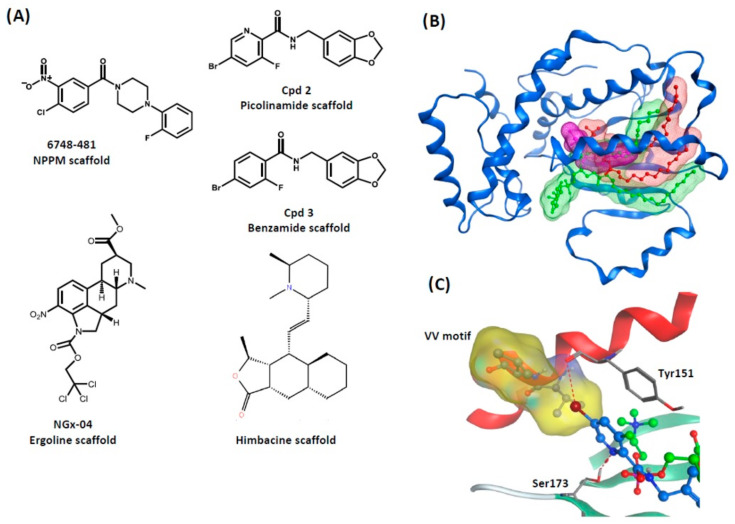
Sec14 small molecule inhibitors. (**A**) Validated small molecule inhibitors of yeast Sec14 are shown. All directly inhibit Sec14 activity in vitro and all but himbacine show fungicidal activities against *S. cerevisiae* in vivo. The picolinamide and benzamide derived inhibitors are termed compounds 2 and 3, respectively, as per the nomenclature used to describe them in [141]. (**B**) Binding pose of picolinamide-based compound 2 (magenta) as determined by X-ray crystallography confirms the Sec14-active small molecule inhibitors occupy the acyl chain binding regions of PtdIns (green overlay) and PtdCho (red overlay) as well the the PtdCho headgroup coordinating region. The Sec14 lipid binding domain is rendered as blue ribbon. (**C**) a magnified view of an overlay of the picolinamide-based inhibitor compound 2 (blue, ball&stick rendering) and PtdCho (green, ball&stick) with respect to VV motif and other PtdCho headgroup-coordinating residues within the Sec14 lipid-binding pocket. The VV-motif is shown in yellow translucent surface render. The halogen bond between the bromine moiety of compound 2 and the backbone keto group of residue Tyr151 is highlighted.

Candidate inhibitors of *S. cerevisiae* Sec14 were identified by chemogenomic screens that interrogated ~3000 inhibitors of yeast growth in a haploinsufficiency profiling (HIP) platform of essential genes, coupled with homozygous profiling (HOP) of non-essential genes [146,147]. Foundational work in this arena came from the validation of nitrophenyl(4-(2-methoxyphenyl)piperazin-1-yl)methanones (NPPMs) as direct and specific inhibitors of Sec14 [114] (Figure 6A). That study established Sec14 PITPs as new targets for interfering with PtdIns(4)P signaling and that such interference could be imposed with exquisite selectivity. Moreover, it set the template by which Sec14-like PITP inhibitors are to be characterized and validated.

Subsequent chemical screens identified small molecules based on ergoline, benzamide, and picolinamide scaffolds that were validated as Sec14-directed small molecule inhibitors [50,117] (Figure 6A). For purposes of simplicity, we will consider the benzamide and picolinamide scaffolds together as picolinamides. It is remarkable how closely the ergolines and picolinamides follow the NPPM rules for mechanism of Sec14 inhibition—as discussed below. Moreover, ergoline-based Sec14 inhibitors show anti-fungal activity with no obvious toxicity to mammalian cells. One such ergoline (NGx04), and its analogues, inhibit growth of clinical isolates of *C. neoformans* and of fluconazole resistant *C. neoformans* [50]. These developments argue that Sec14 inhibitors are suitable lead compounds for development of clinically useful anti-fungal drugs. Moreover, it is also clear that Sec14 is susceptible to inhibition by other unrelated chemical compounds [147]. For example, himbacine is a natural product derived from the bark of the Australian magnolia (*Galbulimima belgraveana*) that was initially investigated as a lead compound for Alzheimer’s disease therapy [148] (Figure 6A). This alkaloid potently inhibits Sec14 activity in vitro but has little fungicidal activity against yeast in vivo for reasons that have not been further investigated (our unpublished data). Nevertheless, those results implicate alkaloid scaffolds as potentially useful starting points for development of new Sec14-directed inhibitors. Because NPPMs and picolinamides represent the most potent Sec14 inhibitors, the remainder of the discussion will focus largely on these two classes of molecules.

## 12. Mechanisms of Sec14 Inhibition and the PtdCho Headgroup Binding Site

The Sec14 PITP is the sole essential NPPM target in yeast, and even yeast PITPs that are close structural paralogs of Sec14 are impervious to NPPM-mediated inhibition in vitro and in vivo growth assays. The relevance of those two readouts to phosphoinositide signaling is also clear, as demonstrated by comprehensive analyses of Sec14-dependent membrane trafficking pathways in NPPM-challenged yeast cells [114]. Upon intoxication of yeast cells by NPPMs, only Sec14-related PtdIns(4)P signaling is impaired—even as PtdIns(4)P signaling pathways aligned with other PITPs remain unaffected. The ability of NPPMs to discriminate between phosphoinositide pools is a direct result of their remarkable specificity towards Sec14 [114]. This specificity is recapitulated for the picolinamides when assessed by yeast growth assays and in vitro Sec14 activity measurements [117]. All available evidence suggests that NPPMs and picolinamides load into the Sec14 hydrophobic pocket during a phospholipid exchange cycle and occlude it in what is an essentially irreversible manner. NPPMs and picolinamides do so by occupying PtdIns and PtdCho acyl chain space within the lipid-binding pocket [114,117] (Figure 6B).

Combined in silico docking and mutagenesis data indicate that a major factor in setting NPPM binding specificity is the polar A-ring of the NPPM molecule. This chemical group engages the Sec14 residues essential for coordination of the PtdCho headgroup, and structural data reveal this basic strategy is recapitulated by picolinamides (Figure 6C). The PtdCho headgroup-coordinating region of the Sec14 lipid binding pocket represents a curious hydropathic microenvironment within a larger hydrophobic environment [110]. As such, this microenvironment is ideally suited for supporting interactions with Lipinsky “rule-of-five” small molecules. The data indicating NPPMs engage Sec14 pocket residues essential for coordinating the PtdCho headgroup account for the indifference of other yeast Sec14-like PITPs (e.g., Sfh2, Sfh3, Sfh4, and Sfh5) to inhibition by NPPMs. Those PITPs are not PtdCho-binding proteins as these do not exhibit a PtdCho-binding barcode. Consequently, these Sec14 paralogs lack the elements required for coordinating NPPM-binding [114], or picolinamide- or ergoline-binding [50,117]. Thus, the PtdCho-binding barcode serves as one of two primary structure signatures that reliably forecasts whether an uncharacterized Sec14-like PITP will be sensitive or resistant to inhibition by the presently characterized Sec14-directed small molecules. By this criterion, there are no Sec14-like proteins/domains expressed by mammals that carry a PtdCho-binding barcode—suggesting no mammalian Sec14-like protein will be inadvertently targeted by NPPM- or picolinamide-based inhibitors of fungal Sec14 PITPs.

## 13. The Sec14 VV-Motif Drug Sensitivity Signature

The second signature that reliably predicts whether an uncharacterized Sec14-like PITP will be sensitive or resistant to inhibition by Sec14-directed NPPMs, ergolines, and picolinamides is the VV-motif that was recognized in genetic screens interrogating mechanisms by which Sec14 acquires NPPM resistance [114]. The motivation for those studies was grounded in the interesting conundrum presented by the Sec14-like PITP Sfh1 regarding the basis of NPPM target-specificity. The Sfh1 PITP is not only highly homologous to Sec14, but Sfh1 also precisely conserves the functional PtdCho-binding motif critical for NPPM and picolinamide binding. However, Sfh1 is intrinsically resistant to NPPM- and picolinamide-mediated inhibition [114,115,117].

The genetic screen of Khan et al. (2016) not only confirmed previously described Sec14 residues critical for NPPM binding, it also identified two previously unappreciated residues (V_154_V_155_) as critical for NPPM binding. Those residues define the VV-motif and are not conserved in Sfh1. However, transplacement of the VV-motif into Sfh1 endows NPPM-sensitivity to that reconfigured PITP. Other VV-motif transplacement experiments extend that observation to NPPM-resistant Sec14 PITPs from other fungal species with divergent VV-motifs—including virulent species such as *C. albicans* [115]. Subsequent studies confirmed this concept also applies to picolinamide-based Sec14 inhibitors [117].

While in silico NPPM modeling experiments suggested that the VV-motif indirectly influences the NPPM-binding substructure [115], the Sec14::picolinamide structure demonstrates the VV-motif is positioned adjacent to the PtdCho headgroup-coordinating unit, and that it directly coordinates bound picolinamide [117] (Figure 6C). Those structural data report that incorporation of larger sidechains at this position imposes a steric incompatibility with picolinamide binding. As minor adjustment in the NPPM dock pose proposed by Nile et al. (2014) is sufficient to bring the VV-motif in immediate proximity of bound NPPM, it is likely that a direct coordination mechanism will apply for NPPMs as well.

## 14. PtdCho Barcode and VV-Motif Conservation in Sec14 Proteins across the Phylogeny of Fungal Pathogens

Although this review features *Candida* as a model fungal pathogen with regards to development of Sec14-directed small molecule inhibitors (with mention of *Aspergillus*, *Cryptococcus,* and *Pneumocystis* species as particularly devastating to immune-compromised individuals [29]), the Center for Disease Control also lists *Histoplasma*, *Coccidioides*, *Blastomyces*, *Mucor*, and others to be of significant infectious disease relevance. Infections involving these pathogenic fungi include mucormycosis of adult brain and lung as well as a sporotrichosis of skin and lung [149]. Newborns are susceptible to fungal infections, ranging from superficial rashes caused by *Trichophyton* and *Candida* to the more serious gastrointestinal infections by *Rhizopus* or *Mucor* species [150,151]. Thus, the spectrum of pathogenic fungal species is expansive. This fact raises the question of just how widely applicable a Sec14-directed targeting strategy might prove in development of antifungals that reach across the breadth of the fungal phylogenetic tree.

**Figure 7:**
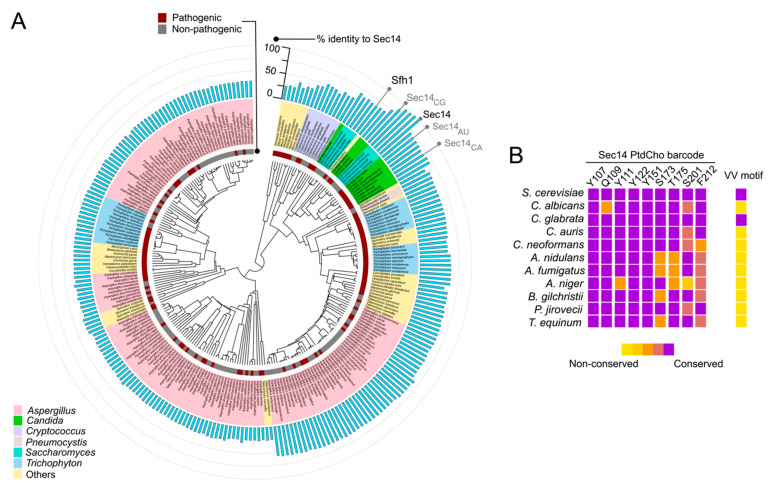
Phylogenetic analysis of fungal Sec14s. (**A**) Phylogenetic analysis of fungal Sec14s. (**A**) Sec14 domain sequences from *Saccharomyces* and major fungal pathogens listed on CDC website (https://www.cdc.gov/fungal/diseases/index.html) were compiled from the Ensembl fungal database (https://fungi.ensembl.org/index.html). The alignment file was processed in JalView to eliminate redundancy and includes only entries with 200 to 800 amino acids. Percent identity to *S. cerevisiae* Sec14 was calculated by pair-wise alignment of each entry using BLAST (https://blast.ncbi.nlm.nih.gov/Blast.cgi). A multiple sequence alignment was performed with the processed sequences using NGPhylogeny.fr portal and the MAFFT 7.4 algorithm [152]. The phylogenetic tree was visualized using the iTol website (https://itol.embl.de) [153] annotated with a bar graph showing percent identity of each entry to *S. cerevisiae* Sec14. Maroon strip indicates virulent fungi, and the grey strip represents non-pathogenic fungi. Fungi are color coded according to genus as indicated. Percent identity of each Sec14-like domain to *S. cerevisiae* Sec14 is indicated by bar graph (blue). The bars corresponding to Sec14 homologs from *C. glabrata* (Sec14_CG_), *C*. *auris* (Sec14_AU_), and *C. albicans* (Sec14_CA_) are labeled in grey. *S. cerevisiae* Sec14 and its close paralog Sfh1 are labeled in black. (**B**) Heat-map depicting the conservation of key PtdCho binding residues and VV-motif in Sec14 orthologs from the following ten virulent fungal species are shown: *C. albicans*, *C. glabrata*, *C. auris*, *C. neoformans*, *A. nidulans*, *A. fumigatus*, *A. niger*, *B. glichristii*, *P. jirovecii,* and *T. equinum*. Accessed on 16 June 2021.

The sensitivities of Sec14 PITP of fungal pathogens to NPPMs and picolinamides can be initially interrogated by assessing conservation of the two signatures of sensitivity—the PtdCho barcode and the VV-motif. That information is summarized in Figure 7. The phylogenetic data illustrate not only the high level of Sec14 conservation across the fungal kingdom on the basis of primary structure, but also highlight conservation of the PtdCho-binding barcode throughout the kingdom. By contrast, the VV-motif is highly divergent—some virulent fungi preserve this motif (e.g., *C. glabrata*), but the large majority do not (including *C. albicans* and *C. auris*). Indeed, we find the specificity with which the VV-motif is altered in Sec14 orthologs of virulent fungal species to be a matter of interest. This observation suggests these fungi encounter some host natural product(s) with inhibitory activity against Sec14, and that these pathogens reconfigured the VV-motif to preserve the functionally essential PtdCho-binding/exchange activity in the face of compromised inhibitor binding. Circumventing this resistance mechanism poses a major challenge in the design of next-generation Sec14-directed inhibitors.

## 15. Pharmacophore and Future Design Considerations

The discovery of SMIs directed against Sec14 PITPs, when coupled with extensive structure-activity relationship (SAR) and mutagenesis approaches, informs strategies for development of next-generation inhibitors. Exploitation of commercially available compounds produced around the NPPM scaffold enabled detailed SAR analyses of the requirements for SMI activity against Sec14. Those SAR data are consistent with both structure-based and unbiased mutagenesis approaches directed towards identifying Sec14 residues required for NPPM and picolinamide binding [114,115,117]. What is remarkable is how closely the NPPM, ergoline, and picolinamide SAR and mutagenesis profiles converge. These inhibitors uniformly target the hydropathic micro-environment that forms the PtdCho headgroup coordinating region of the Sec14 lipid-binding cavity. Given the availability of reasonable docking models for NPPM binding [114] and a crystal structure for the open Sec14 conformer bound to picolinamide and ergoline [117], a structural superposition of NPPM binding onto the Sec14: picolinamide structure identifies common pharmacophoric points for optimizing SMI potency. Design of next-generation NPPM- and picolinamide-based Sec14-directed SMIs guided by this information will fall into two phases.

**Figure 8:**
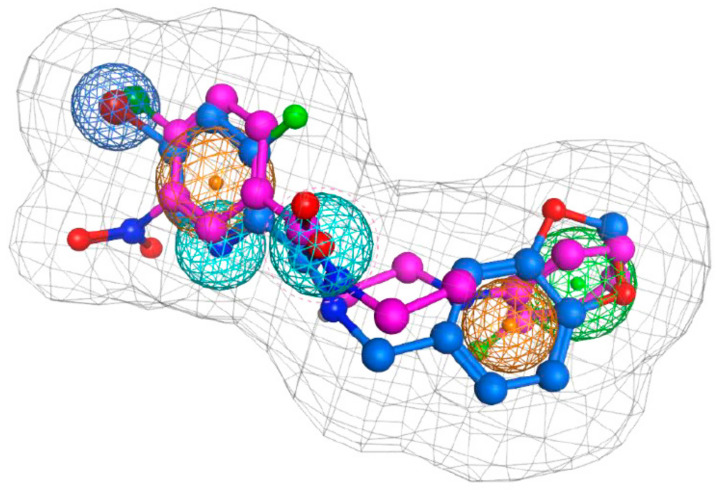
Sec14 Small molecule inhibitor pharmacophore. A Sec14 pharmacophore that describes key features of inhibitor binding to Sec14 is presented. This profile is based on detailed analyses of inhibitors derived from NPPM (magenta) and picolinamide (blue) scaffolds. The pharmacophore features a halogen bond center (blue meshed sphere), aromatic centers (orange meshed sphere), H-bond acceptors (cyan meshed sphere), hydrophobic centers (green meshed sphere), and ligand volume (grey mesh covering the ligand volume).

The first phase will involve modulating the chemical properties of the available scaffolds to improve potency and to effectively substitute pharmacologically undesirable chemical groups. The composite NPPM/picolinamide-defined pharmacophore identifies a hydrophobic planar ring system (the A-ring) that acts as a scaffold to properly position the essential halide functional group for interaction with Sec14 (Figure 8). Incorporating pharmacologically more palatable electron-withdrawing substitutions for the highly reactive nitro-moiety at the meta-position of the A-ring promises the dual benefits of reducing potential toxicities associated with the nitro-moiety while maintaining NPPM potency. With regard to the remainder of the scaffold, the distal planar ring system (B-ring) adopts a conformation favorable for docking to the amphipathic region of the binding cavity. This configuration in the Sec14 binding pocket offers opportunities for rational modification of the B-ring for optimization of binding affinity.

**Figure 9:**
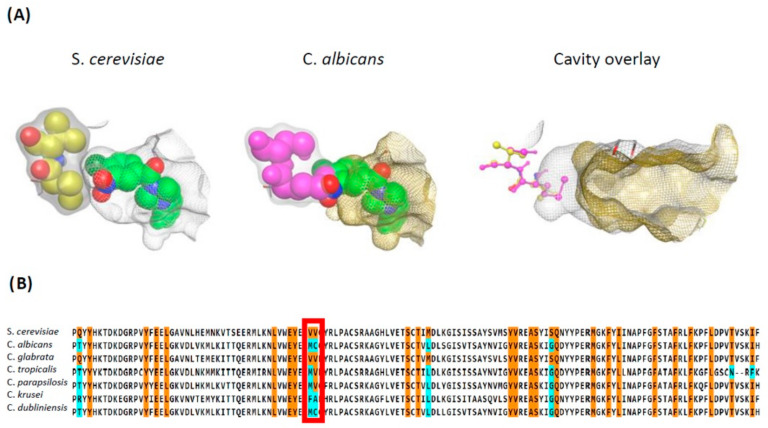
VV-Motif and Sec14 resistance/sensitivity to validated small molecule inhibitors. (**A**) The VV-motif and polymorphisms and the associated structural differences are highlighted in comparison of the NPPM binding space within the lipid binding pockets of *S. cerevisiae* and *C. albicans* Sec14 PITPs. Left panel: The *S. cerevisiae* binding pocket is shown with the dock pose of bound NPPM illustrated in green space fill render. The VV-motif is highlighted in yellow space fill representation, and the lipid binding pocket surface is rendered as transparent grey mesh. Center panel: The *C. albicans* binding pocket is shown with a dock pose of NPPM rendered in green space fill, and the lipid binding pocket surface is rendered as transparent gold mesh. The *C. albicans* binding VV-motif cognate is diverged (MC), and this MC-motif is shown in magenta space fill representation. The steric clash between the MC-motif and the NPPM binding space is apparent. Right panel: The VV-motif regions of the *S. cerevisiae* and *C. albicans* Sec14 lipid binding pocket are compared. The *S. cerevisiae* VV-motif (yellow ball&stick) and the cognate *C. albicans* MC-motif (magenta ball&stick) residues are highlighted, and the lipid binding pocket surfaces of *S. cerevisiae* (grey) and *C. albicans* (gold) are rendered in transparent mesh. Note the *C. albicans* pocket space is contracted relative to that of S. cerevisiae because of the VV–MC polymorphism and this contraction prohibits accommodation of the aryl halide warheads of NPPM and picolinamide-based Sec14 inhibitors. (**B**) Residues of the lipid binding domain regions that form the lipid binding cavities of Sec14 PITPs from *S. cerevisiae* and the indicated *Candida* species are aligned. Residues whose sidechains line the lipid binding pocket surface are highlighted in orange, and polymorphisms at those positions are identified in cyan. The VV-motif is highlighted by the red box.

The second phase will involve redesigning these scaffolds so that the next generation compounds are able to target Sec14 PITPs that harbor a relaxed VV-motif—as is the case with most virulent fungi. The pathogens expressing Sec14 PITPs with diverged VV-motifs, such as *C. albicans*, *C. auris,* and others, exhibit a contracted binding pocket that cannot accommodate the A-ring of NPPMs or picolinamides (Figure 9). Thus, whereas the distal regions of NPPMs and picolinamides remain effective, the proximal A-ring system needs to be comprehensively redesigned to circumvent those steric problems. Although there are no crystal structures available for Sec14 PITPs of pathogenic fungi, these proteins share such high primary structure identity with *S. cerevisiae* Sec14 (and with Sfh1) that suitable structural templates are available. Thus, in silico-based strategies that constrain docking by demanding preservation of key pharmacophoric interactions represent promising first approaches.

**Figure 10:**
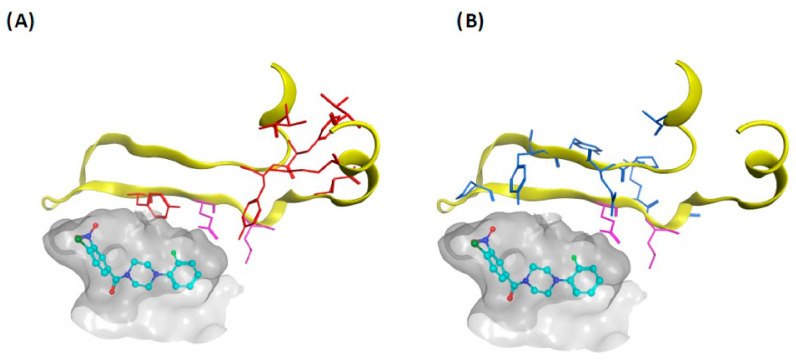
Mapping drug sensitivity and resistance. Panel highlights the results from a targeted window of CRISPR/Cas9-based saturation editing mutagenesis. (**A**) Individual residues where specific missense substitutions increase Sec14 sensitivity to inhibition by NPPM are highlighted in red sticks. (**B**) Residues where specific missense substitutions increase Sec14 resistance to NPPM are highlighted as blue sticks. Residues where missense substitutions can increase both sensitivity or resistance as a function of the specific substitution are highlighted as magenta sticks. NPPM is rendered in cyan ball-and-stick mode within the grey translucent binding pocket. Saturation editing mutagenesis was executed in the window bounded by Sec14 residues 102-137 (highlighted as yellow ribbon).

While the PtdCho barcode and the VV-motif are useful predictors of fungal Sec14 susceptibility to NPPMs and picolinamides, this information may yet prove insufficient for confident profiling of Sec14s and rational drug design on the basis of primary structure alone. Recent proof-of-concept deployment of a new CRISPR/Cas9-based method for saturation editing of yeast genes outlines prospects for more complete and unbiased assessments of Sec14:NPPM interactions and demonstrates there is much to be learned in this regard [154]. Saturation editing of Sec14 residues 102–137, where every amino acid residue was systematically altered to each of the other nineteen amino acids, coupled with assessment of the NPPM sensitivity of each variant, accurately recapitulated previous findings that residues Y_111_ and Y_121_ of the Sec14 PtdCho-binding barcode are critical for NPPM binding. Interestingly, considering the narrow window interrogated, the analysis identified not only new hotspots for NPPM resistance but also identified hotspots for increased NPPM sensitivity [154] (Figure 10). Expansion of this approach to a survey of the entire Sec14 primary structure, and interrogation of other SMI scaffolds in a comprehensive set of comparative analyses, promises to deliver a high-resolution picture of the landscape of Sec14:drug interactions, specifically, one superior to those that can be gleaned from application of conventional genetic and variomic drug resistance screens or from structural analyses of Sec14: inhibitor co-complexes.

## 16. A Sec14-Directed Natural Product with Potent Anti-Fungal Activity?

The suitability of Sec14 as a druggable target is reinforced by the results of an unbiased natural product screen for antifungals directed against virulent fungi where Sec14 once again emerges as a likely cellular target. Metabolomic survey of marine microbiomes identified turbinmicin, a secondary metabolite isolated from the *Micromonospora* bacterium that contributes to the microbiome of a sea squirt *Ecteinascidia turbinata*, as a new class of Sec14-directed inhibitor [155]. Turbinmicin is a broad-spectrum polyketide with growth inhibitory activity against most virulent fungal species (including derivatives resistant to echinocandins and azoles and pan resistant *C. auris*) that: (i) shows little toxicity to human red blood cells and mice at concentrations in large excess of effective antifungal concentrations and (ii) reduces fungal load in an immunocompromised disseminated *C. auris* and *A. fumigatus* mouse models [155,156].

Chemogenomic analyses in *S. cerevisiae* of the type used previously again implicated Sec14 as a primary cellular target for turbinmicin, and one line of indirect experimental support for this assignment was culled from imaging assays that demonstrated turbinmicin intoxication inhibited membrane trafficking from the TGN/endosomal system—a known execution point for Sec14 function [155]. Inhibition of this execution point translates to consequences for the virulent fungus as turbinmicin inhibits biofilm formation by interfering with the delivery of extracellular vesicles crucial for biofilm assembly [156]. However, it remains to be demonstrated that Sec14 is the direct and the sole essential turbinmicin target (by the criteria set forth by Nile et al. [114]), or that turbinmicin itself is the active antifungal compound rather than a pro-drug that is subsequently metabolized to the active inhibitor by the fungal cell. Nevertheless, the turbinmicin discovery potentially extends the chemical space for further rational design of Sec14-directed inhibitors. Notably, turbinmicin exhibits effective antimicrobial and biofilm-disrupting properties against *C. glabrata* (whose Sec14 PITP preserves the VV-motif) and *C. albicans*, *C.auris,* and *A. fumigatus* (whose Sec14 PITPs present diverged VV-motifs) [154,156]. As a result, if this structurally complex polyketide directly targets Sec14, it does so in a unique manner that differentiates it from NPPM, ergoline, and picolinamide scaffolds in that its mechanism of inhibition is indifferent to status of the VV-motif. The turbinmicin discovery also serves as a firm reminder that natural product screening approaches are not to be ignored. For example, fungi and actinomycetes compete for ecological niches and, as mentioned above, amphotericin B is a secondary metabolite produced by soil actinomycetes. Natural product screens for actinomycete secondary metabolites that inhibit Sec14 have not, to our knowledge, been pursued, and these might well bear significant fruit.

## 17. Sec14-Directed Anti-Fungals and Prospective Mechanisms of Drug Resistance

To what extent will the emergence of drug-resistant pathogens reduce the efficacy of any Sec14-directed antifungal drug? Fungi deploy a number of mechanisms to thwart the action of antimycotics. However, here too, Sec14-directed inhibitors potentially offer some advantages. The two general pathways for resistance involve evolution of SNPs that either modify the SMI binding site so that the inhibitor binding is impaired (intragenic mechanism that is a genetically dominant trait) or that enable some indirect (i.e., extragenic) mode that either facilitates drug efflux from cells or increases expression of the target so that pharmacologically effective levels of drug are increased. Such extragenic mechanisms can describe either genetically dominant or recessive traits. Emergence of fungi resistant to Sec14-directed anti-fungals will include both intragenic and extragenic mechanisms. However, much is known about the mechanisms by which cells can become resistant to Sec14-targeted SMIs in *S. cerevisiae*, and that information provides insights into potential resistance mechanisms. Moreover, those data forecast that such mechanisms will come with a penalty to the organism.

Naturally occurring intragenic mutations that preserve Sec14 activity while impairing NPPM binding were characterized in a genetic screen for spontaneous NPPM resistance in the *S. cerevisiae* model [115]. Those missense mutations resulted in single amino acid substitutions to the Sec14 protein sequence that corresponded to the lipid binding pocket and included residues that interact directly with NPPM. The localization of those alterations to the lipid binding pocket resulted in attenuated Sec14 lipid exchange activities—i.e., these came with functional consequences [115]. As such, these resistance mutations might be expected to attenuate fitness of the fungal pathogen and, therefore, its virulence. Single base-changes altering the VV-motif (included in the screen where V→F alterations were identified; [115]) also identify an obvious mechanism for acquiring resistance, but the spectrum of such mutations (assuming single base changes) is predictable. As a result, the corresponding mutant proteins can be targeted in advance by rational inhibitor design. The spectrum of Sec14 missense substitutions that confer NPPM resistance also inform mechanisms of resistance to ergolines and picolinamides. However, should Sec14 prove to be the direct target of turbinmicin, the spectrum of Sec14 resistance mechanisms might overlap but will no doubt differ in other key respects.

**Figure 11:**
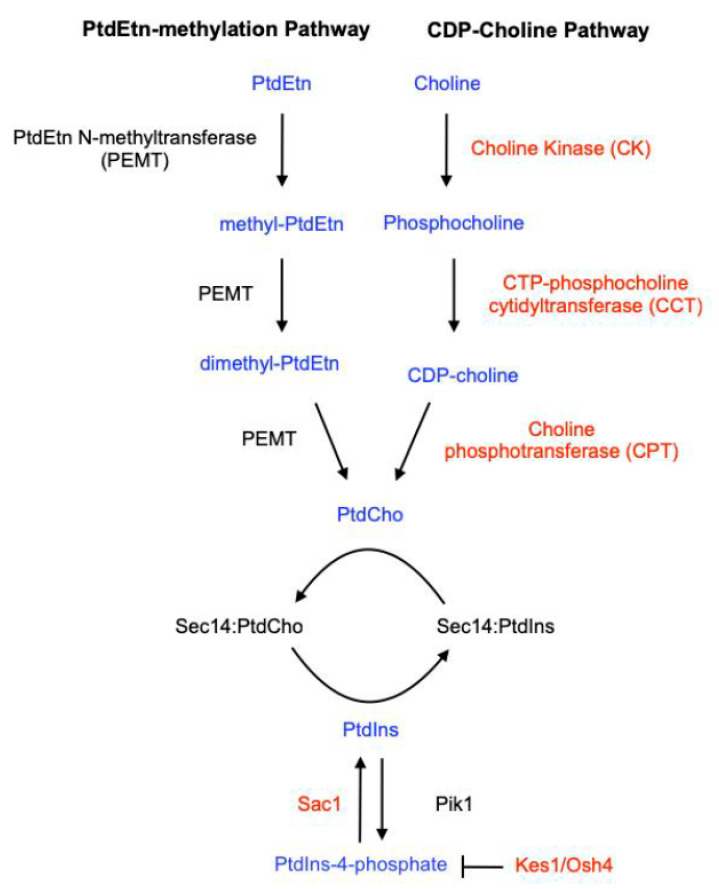
Mechanisms for “bypass Sec14”. Loss-of-function mutations in specific non-essential genes restores cell viability in the face of functional ablation of the normally essential Sec14. These “bypass Sec14” mutations affect the individual metabolism of both phospholipid binding ligands of Sec14. The affected pathways are shown in simplified form. Mutations that affect PtdCho metabolism specifically inactivate the CDP-choline pathway for PtdCho biosynthesis, the enzymes of which are highlighted in red. Interestingly, ablation of the de novo phosphatidylethanolamine (PtdEtn) methylation pathway for PtdCho biosynthesis, even under conditions where this pathway is the sole pathway for net production of PtdCho, has no effect on the cellular requirement for Sec14 function. Loss-of-function mutations that impact inositol phospholipid metabolism do so via inactivation of antagonists of PtdIns(4)P signaling. These include mutations that functionally ablate the Sac1 PtdIns(4)P phosphatase Sac1 and or the PtdIns(4)phosphate/sterol exchange protein Kes1/Osh4—also highlighted in red.

In *S. cerevisiae*, extragenic mechanisms for bypassing the essential Sec14 requirement for cell viability and membrane trafficking have also been described and analyzed. Interestingly, these all describe recessive loss-of-function mutations that either: (i) inactivate the CDP-choline pathway for PtdCho biosynthesis [132], (ii) inactivate the Sac1 PtdIns(4)P phosphatase [67,71,157,158], or (iii) inactivate the Kes1/Osh4 ergosterol/PtdIns(4)P exchange protein [159,160,161] (Figure 11). As these are recessive traits, their occurrence requires “two-hits” in diploid pathogens and will occur rarely. Moreover, studies in *S. cerevisiae* project that these mechanisms come with their own burden. Growth of Sac1-deficient cells is severely attenuated, even under laboratory conditions [157,158]. Kes1/Osh4 mutants exhibit a deregulated cell cycle that will likely compromise fitness under stress conditions [133,135]. What penalty might be associated with loss of a major pathway for synthesis of PtdCho (a major bulk phospholipid) remains to be determined. However, such a deficit is likely to prove detrimental to an infecting pathogen undergoing the high-capacity metabolic reprogramming associated with dimorphic transition to a mycelial growth mode and to the demands associated with the formation and maintenance of biofilms.

Finally, one mechanism for drug resistance involves increased expression of ABC transporters that prevent intracellular accumulation of the antifungal drug. However, small molecule inhibitors are being developed that block activity of the transcription factors required for expression of such efflux pumps [9,162]. Thus, combination therapies remain a viable option in instances where resistance to a Sec14-directed anti-mycotic is the result of elevated expression of ABC transporters that export drug from the cytoplasm to the extracellular environment.

## 18. Conclusions

Effective development of antifungals defines an urgent need highlighted by erosion in the efficacies of the three classes of workhorse antifungals that have been historically deployed. This effort will require invention of sophisticated new approaches for intervening with familiar fungal targets and creative approaches for identifying and targeting new ones. Fungal Sec14 PITPs represent an example of the latter. These proteins are highly discriminating portals through which chemical inhibition of fungal phosphoinositide signaling can be executed with exquisite specificity. At least four distinct chemical scaffolds have already been identified from which Sec14-directed inhibitors can be built, and there is now sufficient information to guide rational development of next-generation anti-Sec14 small inhibitors directed against Sec14 PITPs of virulent fungi. In that regard, the recent turbinmicin discovery suggests yet another promising path towards achievement of this goal. As Sec14 PITPs are either essential for cell viability or essential for processes required for fungal virulence, such compounds hold substantial promise as a source of a new generation of novel drugs for combating the escalating problem of fungal infectious disease.
